# Neurocognitive function in lower grade glioma patients selected for proton radiotherapy: real-world data from a prospective cohort study

**DOI:** 10.1007/s11060-025-04973-7

**Published:** 2025-02-20

**Authors:** Hiska L. van der Weide, Anne M. Buunk, Femke F. Siebenga, Johannes A. Langendijk, Agata Bannink-Gawryszuk, Ingeborg Bosma, Roelien H. Enting, Anouk van der Hoorn, Hanne-Rinck Jeltema, Michiel Wagemakers, Rob J. M. Groen, Annemiek M. E. Walenkamp-Hageman, Janine Nuver, Miranda C. A. Kramer, Jacoba M. Spikman

**Affiliations:** 1 Department of Radiation Oncology, University of Groningen, University Medical Center Groningen, Groningen, The Netherlands; 2 Department of Neurology, Unit of Neuropsychology, University of Groningen, University Medical Center Groningen, Groningen, The Netherlands; 3 Department of Neurology, University of Groningen, University Medical Center Groningen, Groningen, The Netherlands; 4 Department of Radiology, University of Groningen, University Medical Center Groningen, Groningen, The Netherlands; 5 Department of Neurosurgery, University of Groningen, University Medical Center Groningen, Groningen, The Netherlands; 6 Department of Neurosurgery, Faculty of Medicine Universitas Airlangga, Dr. Soetomo General Academic Hospital, Surabaya, Indonesia; 7 Department of Medical Oncology, University of Groningen, University Medical Center Groningen, Groningen, The Netherlands; 8 Department of Radiation Oncology, University Medical Center Groningen – University of Groningen, PO Box 30.001, Groningen, 9700RB The Netherlands

**Keywords:** Neurocognitive function, Low grade glioma, Proton therapy, Radiotherapy

## Abstract

**Purpose:**

To determine neurocognitive function (NCF) profiles of patients with lower grade glioma (LGG) eligible to undergo proton radiotherapy (PRT), and how these relate to clinical and radiological characteristics. PRT is offered to those patients for whom sparing of NCF is considered important given their favorable prognosis. To date it is unknown to which extent their NCF profiles are favorable as well.

**Methods:**

A consecutive cohort of 151 LGG patients eligible for PRT according to prevailing Dutch criteria, referred between 2018 and 2023, were assessed with standardized neuropsychological tests prior to PRT. Scores were compared to norm-scores. Composite scores were calculated for the total NCF and 6 separate cognitive domains, and profiles were related to tumor location. Clinical and radiological factors characterizing overall NCF impaired patients were investigated, comparing 3 definitions for impairment.

**Results:**

Patients had on average significantly lower NCF than their norm-group, but interindividual variability was large. For 100/151 patients (66.2%), all cognitive domains were intact, whereas 15/151 patients (9.9%) displayed multiple domain impairments. Poorer NCF was related to right-sided LGG laterality, larger PRT target volume, no Wait & Scan policy, worse neurological function and worse radiological indices (Fazekas and global cortical atrophy, respectively). LGG involvement of the left temporal and occipital lobes was associated with, respectively, lower verbal memory and processing speed.

**Conclusion:**

Prior to PRT, the majority of selected LGG patients display favorable NCF profiles. However, a subgroup showed NCF impairments, with multiple relevant clinical and radiological covariates.

**Supplementary Information:**

The online version contains supplementary material available at 10.1007/s11060-025-04973-7.

## Introduction

Diffuse isocitrate dehydrogenase (IDH) mutated glioma WHO grade 2 and 3, also known as lower grade glioma (LGG), are slowly growing primary brain tumors, with an incidence of approximately 1:100,000 [1]. Patients with LGG have good chances on long-term survival after multimodality treatment, with median survival rates extending beyond 10 years [2–4]. Hence, quality of life is an important focus. The internationally accepted treatment recommendation of maximum safe resection followed by focal radiotherapy (RT) and chemotherapy, can result into treatment-associated neurotoxicity with profound effects on neurocognitive function (NCF) [5]. Impaired NCF has a negative influence on daily functioning and quality of life [6]. Therefore, proton radiotherapy (PRT), a novel RT technology presumed to result in better NCF sparing, is particularly relevant for patients with LGG that is considered favorable according to prevailing criteria.

Changes in NCF in patients with LGG result from a complex interplay between multiple brain damaging factors and intrinsic factors that facilitate adaptation and repair [7, 8]. Over the years, increasing awareness of the relevance of implementing assessment of NCF in clinical studies has resulted in better insights into clinical factors that affect NCF, and changes in NCF over time [5, 9, 10]. The presence and growth of the tumor and its interference with the brain plays an important role. At diagnosis, the majority of (treatment-naïve) patients already show some decline in NCF compared to healthy controls, but not necessarily on an impairment level [11]. Limited evidence suggests that surgery has no substantial additional impact on NCF [12–19]. Still, comprehensive information regarding the cognitive profiles of LGG patients at initiation of RT (with or without an extended period of Wait & Scan policy), in particular regarding which proportion of patients performs at an impaired level, and to which cognitive domains this applies, is lacking. We deem it imperative to obtain a clear overview of neurocognitive status and variability in cognitive profiles at baseline for LGG patients eligible for PRT, as this is essential for assessing its effectiveness in preserving NCF in the long-term. In particular it is relevant to know whether the overall classification of favorable, giving LGG patients access to PRT, also applies to their neurocognitive status.

The aim of this work is to provide a comprehensive description of the NCF of patients at initiation of PRT. We present baseline neurocognitive data that was prospectively obtained in a large cohort of LGG patients eligible for PRT because of their favorable prognosis. Furthermore, we wanted to identify the extent of NCF profiles variability in these selected patients, regarding the extent to which neurocognitive domains were differently affected, and in particular, to investigate whether there were patients who performed overall on an impaired level, comparing different criteria. Finally, we wanted to determine whether relevant clinical and radiological characteristics could be related to specific cognitive profiles, or to an overall impaired profile. The overarching goal of the study was to identify characteristics that might be crucial for future evaluations of the NCF-preserving effects of PRT.

## Patients and methods

All adult (> 18 years) patients with IDH mutated WHO grade 2–3 glioma [20], referred between March 2018 and March 2023 for PRT to the University Medical Center Groningen (UMCG) were eligible for this study. Patients were selected and referred based on nationally defined and accepted eligibility criteria: (1) good clinical condition; (2) favorable prognosis; and (3) dose benefit of protons over photons by means of comparison planning [21]. Patients with juvenile or circumscript LGG (i.e., pilocytic astrocytoma or pleiomorphic xanthoastrocytoma) were excluded.

All neuro-oncological patients treated at UMCG are monitored intensively in a prospective, longitudinal, multidisciplinary clinical registration program [Supplementary Table 1], including assessment of neurocognitive functioning before start of PRT. The program has been reviewed by the medical ethics board of the UMCG, the Netherlands [METc 2017.478; Research Register 201700619]. From all patients included in this study, a written informed consent for use of the data for research purposes was obtained.

### Clinical assessment

Patient’s LGG treatment history, WHO performance score and comorbidity were assessed by the treating radiation oncologist. A policy of ‘Wait & Scan’ was defined as the presence of a LGG treatment-free period of 6 months or longer since diagnosis. In general, wait and scan policy is considered for patients with more favorable features (age, LGG subtype, tumor location and size, tumor resectability, neurological function). Neurological functioning was assessed by a neurologist including the Neurologic Assessment in Neuro-Oncology (NANO, scale 0–23; lower score indicates better function), epilepsy status and current anti-epileptic drug (AED) use.

### Neuropsychological assessment (NPA)

A comprehensive NPA was performed before start or in the first week of PRT by certified (clinical) neuropsychologists experienced in testing neuro-oncological patients. Neuropsychological tests covered 6 cognitive domains, presented in Table 1. Educational level was scored according to a Dutch classification system, ranging from 1 (no primary school) to 7 (university level) [22]. The total time to complete the NPA was approximately 3 h.

**Table 1:**
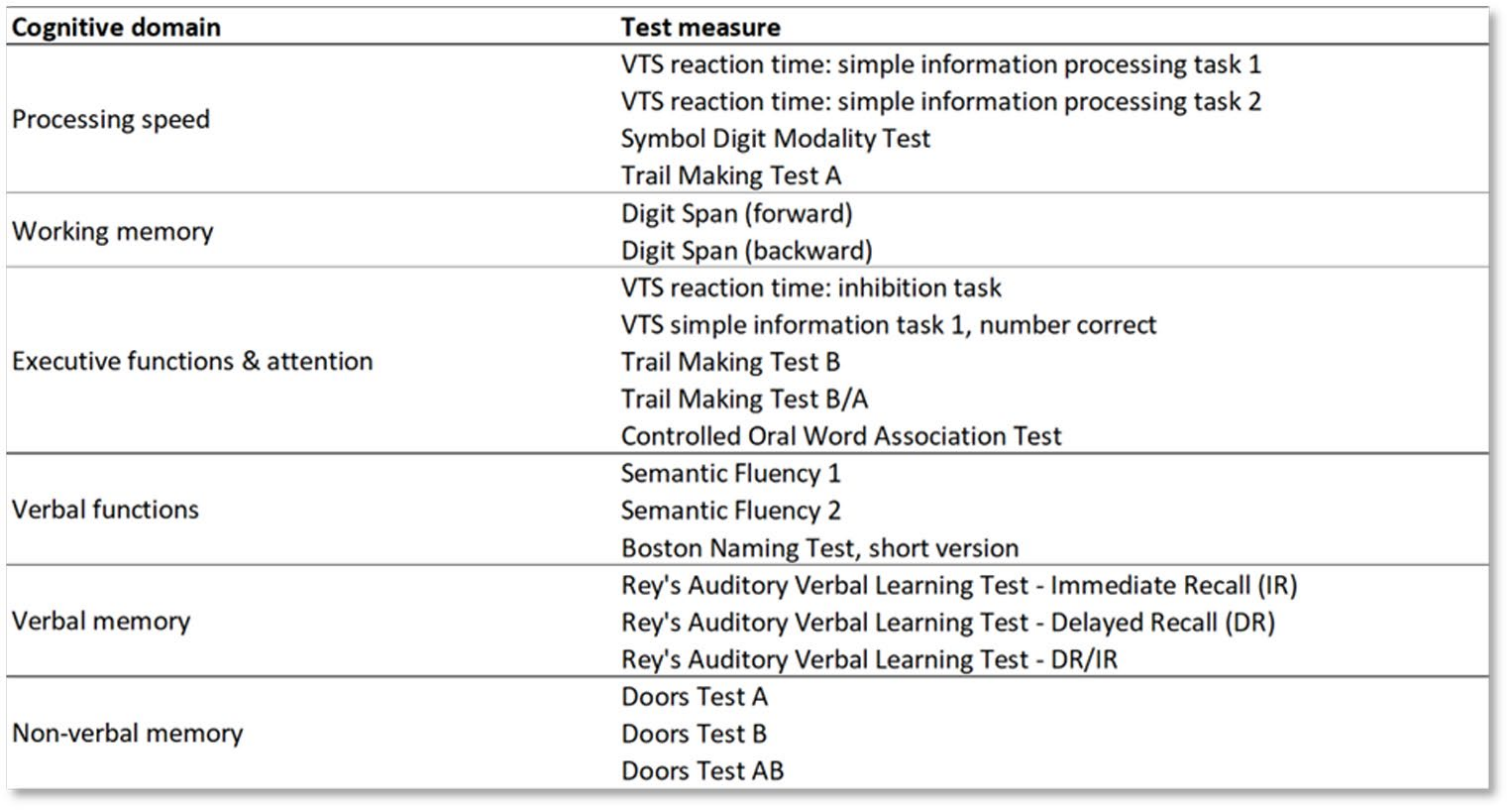
Assessment of cognitive domains and corresponding neuropsychological tests. VTS = computerized Vienna Test System

### MRI imaging and radiological assessment

A PRT planning MRI and CT were performed and co-registered in the planning software (Ray Station, Stockholm, Sweden) and used for target definition. The clinical target volume (CTV) was defined as a 5–10 mm expansion of the tumor-bed and residual tumor on FLAIR. Tumor location (main mass of the tumor), involvement of brain lobes, Fazekas score (0–3; higher score indicates more white matter disease) and Global Cortical Atrophy score (GCA, 0–3; higher score indicates more brain atrophy) were scored by 2 expert neuro-radiation-oncologists (HvdW and MK).

### Statistical analysis

Patient, tumor and treatment characteristics were analyzed using descriptive statistics. Clinical and radiological factors were dichotomized for further analyses: AED use (no vs. yes), NANO (0 vs. 1 or higher), Fazekas (0 vs. 1 or higher) and GCA scores (0 vs. 1 or higher), tumor involvement score per lobe (none vs. any). Raw neuropsychological test scores were transformed into norm-scores (T-score or percentile score) based on Dutch normative data correcting for relevant biographical variables (age, sex, educational level). These norm-scores were converted into Z-scores to enhance comparability.

Composite Z-scores were calculated for the total set of neuropsychological measures (total score) and per cognitive domain (domain score) by calculating the average of the corresponding Z-score measures. In case of missing measures, an average total score of the available measures was used for analysis. For the total score, 3 definitions for (mild) NCF impairment were applied to split the patient cohort in a group with or without NCF impairment: (1) total composite Z-score lower than − 1.0 ^23^; (2) Z-score of < -2.0 on a single measure and/or < -1.5 on multiple measures (according to the International Cognition and Cancer Task Force (ICCTF) recommendations [24], applied to the total set of neuropsychological measures; (3) definition 2 applied to a core set of 3 neuropsychological tests recommended by the European Particle Therapy Network (EPTN) [25]: Rey’s auditory verbal learning test (RAVLT), controlled oral word association test (COWAT) and trail making test (TMT). For the domain score, a single definition of impairment was used: domain composite Z-score lower than − 1.5. The total score and the mean composite domain scores of the group were compared with the expected norm-scores of the healthy population (Z = 0) using a one sample T test.

Between-group comparisons (chi-square, Mann Whitney U) were used to compare clinical and radiological factors of patients with and without NCF impairment. The analyses were conducted in Statistical Package for the Social Sciences, Version 28.0. A p value of < 0.05 (2-sided) was considered significant. Additionally, cognitive domain scores of patients with and without tumor involvement of each brain lobe were analyzed exploratorily, using Bonferroni Holm correction for multiple comparisons.

## Results

160 patients fulfilling the favorable prognosis criteria for PRT were eligible for this study. NCF was assessed in 155/160 patients (96.8%). Reasons for no NPA were language barrier (*n* = 2), COVID-pandemic (*n* = 1), patient’s refusal (*n* = 1) or logistical (*n* = 1). Another 4 patients were excluded because of incomplete NPA (*n* = 4). In total 151/160 patients were available for analysis, resulting in a net compliance rate of 94%.

### Description of the patient cohort

Characteristics of the patients are shown in Table 2. No patients were exposed to intracranial RT prior to PRT. Of the 5 patients that received chemotherapy, 2 patients started PRT directly afterwards (sequential treatment) and 3 patients started PRT at progression (next line treatment). For patients with a Wait & Scan policy in their treatment history, the median interval between diagnosis and start PRT was 23.7 months (inter-quartile range (IQR) 62.1 months).

**Table 2:**
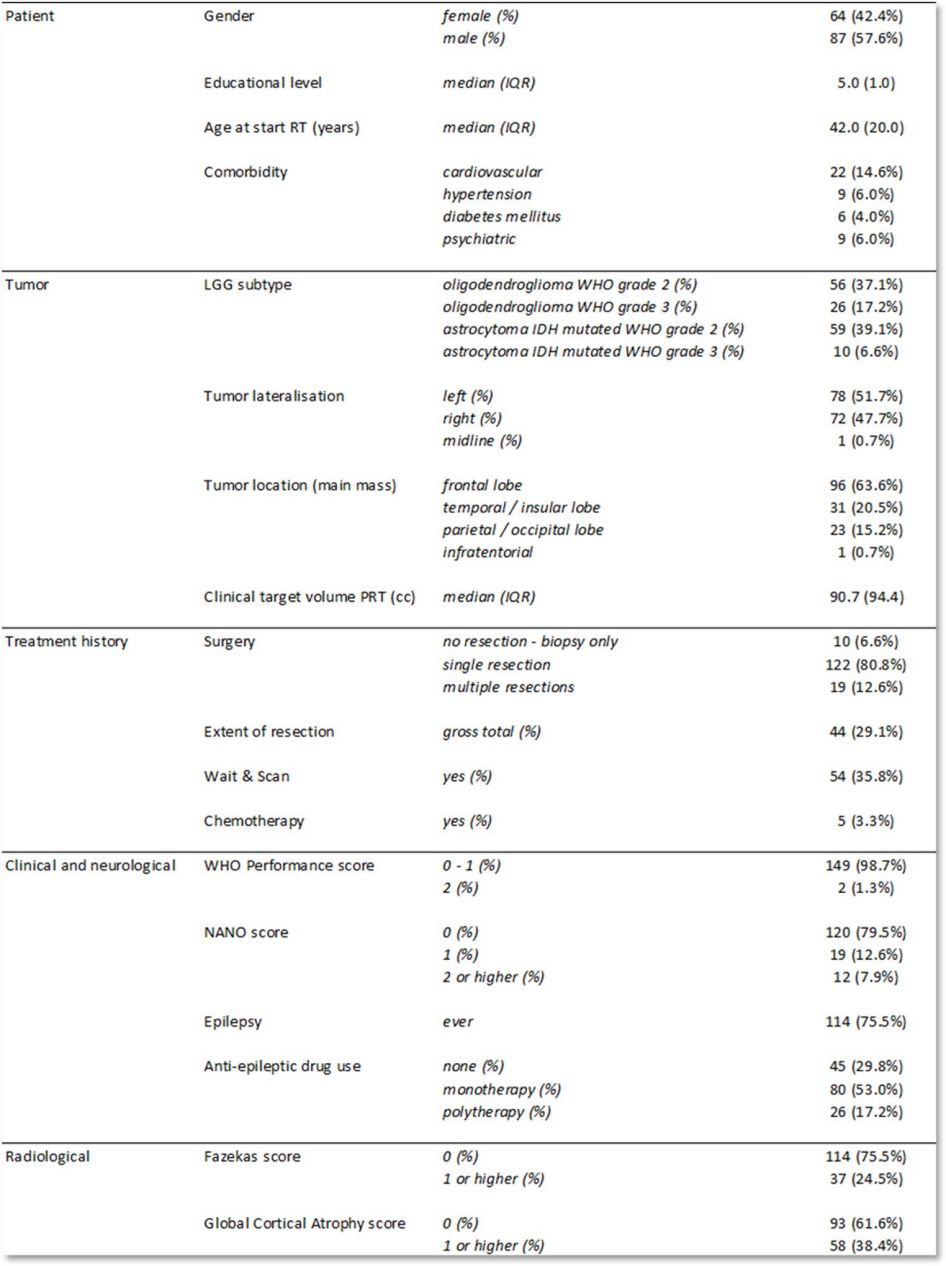
Patient characteristics prior to PRT

### NCF profiles

**Fig. 1:**
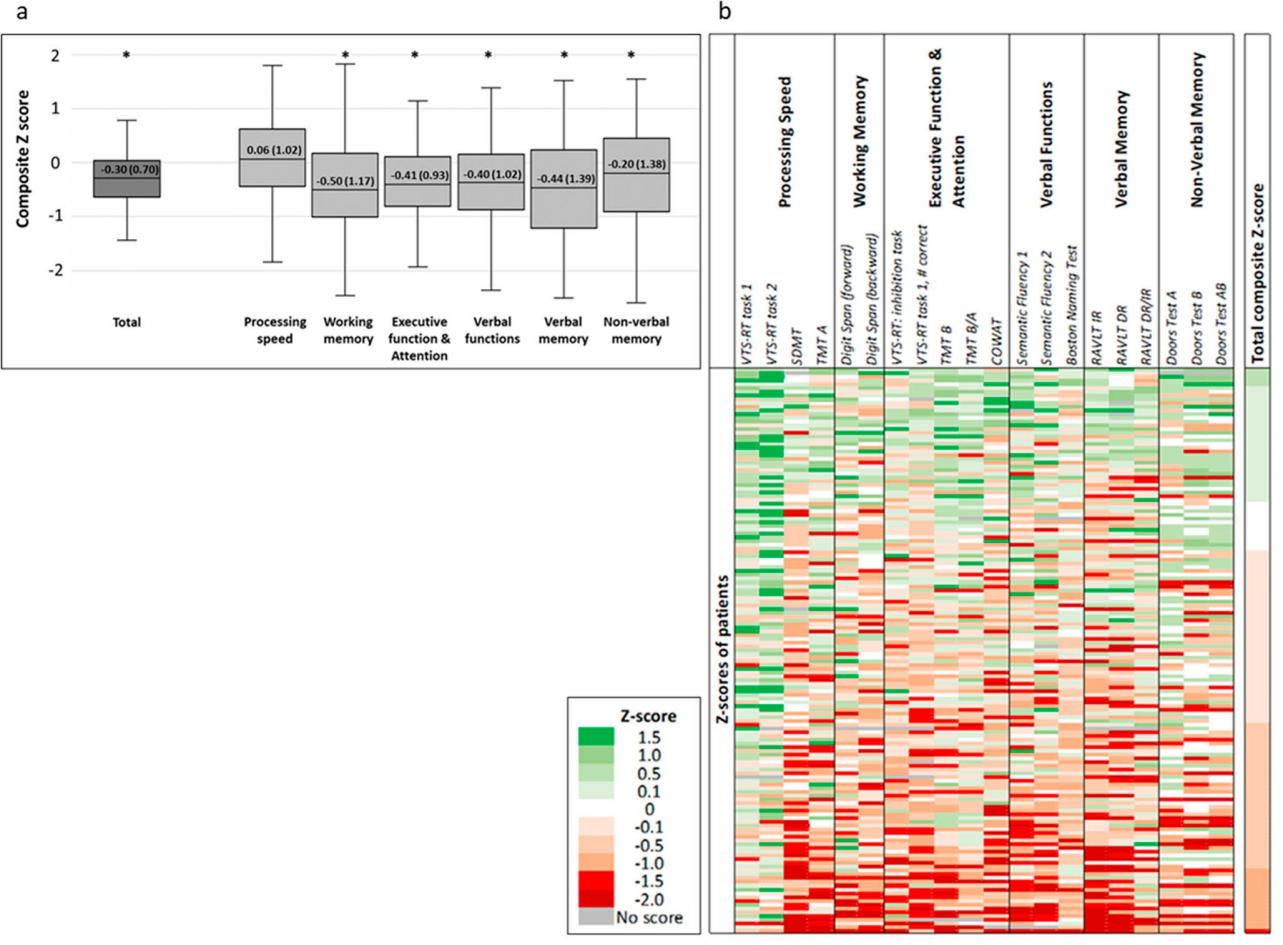
**a** Total and domain NCF composite Z-scores prior to PRT. The boxes represent the interquartile range (IQR) and the whiskers the range, the median is indicated with a horizontal line. The median and IQR values are depicted in the boxes. A Z-score of 0 represents the mean in the normal population, and each unit a standard deviation. An ‘_*_’ indicates a significant difference of the patient cohort from the normal population. **b** Heatmap of individual NCF scores of patients prior to PRT. Each row represents an individual patient, and each column the test measures grouped into cognitive domains as specified in Table 1. Green fields indicate Z-scores higher than 0, and orange-red fields a negative Z-score. Patients are sorted by the total composite Z-score. VTS-RT = Computerized Vienna Test System Reaction Time, SDMT = Symbol Digit Modality Test, TMT = Trail Making Test, COWAT = Controlled Oral Word Association Test, RAVLT = Rey’s Auditory Verbal Learning Test, IR = Immediate Recall, DR = Delayed Recall

The total and domain NCF composite Z-scores for the patient cohort are shown in Fig. 1a. The total score was significantly lower than that of the healthy norm population, but was only lower than − 1.0 in 17 patients (11.3%), including 1 patient (0.6%) with a score lower than − 1.5. Furthermore, in all domains, except processing speed, the mean composite scores were significantly lower than that of the healthy norm population. The percentage of patients with domain scores lower than − 1.5 were: processing speed 2.0%, working memory 7.3%, executive functions and attention 4.1%, verbal functions 8.1%, verbal memory 15.5%, and non-verbal memory 14.0%. There were 100 patients (66.2%) without any domain impairments, 36 patients (23.8%) with 1 domain impairment, and 15 patients (9.9%) with 2 or more domain impairments.

The individual scores of patients are visualized in a heatmap [Fig. 1b]. There was a large variability of performance among patients within the different cognitive domains (intra-domain variability), as well as a large variability across domain scores for individual patients.

### NCF impairment and association with clinical and radiological factors

The number of patients labeled as having (mild) NCF impairment, was highly dependent on the definition of impairment that was applied [Table 3]: definition (1) ‘total Z-score < -1.0’ 17 patients (11.3%); definition (2) ‘ICCTF total set’ 97 patients (64.2%); definition (3) ‘ICCTF core set’ 59 patients (39.1%). Comparing patients with and without NCF impairments, no differences in LGG histology, LGG grade, history of epilepsy, AED use or timing of NPA after last surgery were found, irrespective of the definition applied. The patients with NCF impairment according to definition 1 represented a group with significantly larger PRT target volumes (CTV), more right sided tumor location, worse neurological function (NANO 1 or higher) and more frequent signs of white matter damage on MRI (Fazekas 1 or higher). The patients selected by definition 2 displayed significantly less frequently a treatment history with Wait & Scan and larger PRT target volumes. Selected patients by definition 3 displayed significantly more frequently signs of white matter damage and cortical atrophy. The relations of domain impairments with clinical factors can be found in Supplementary Table 2.

**Table 3:**
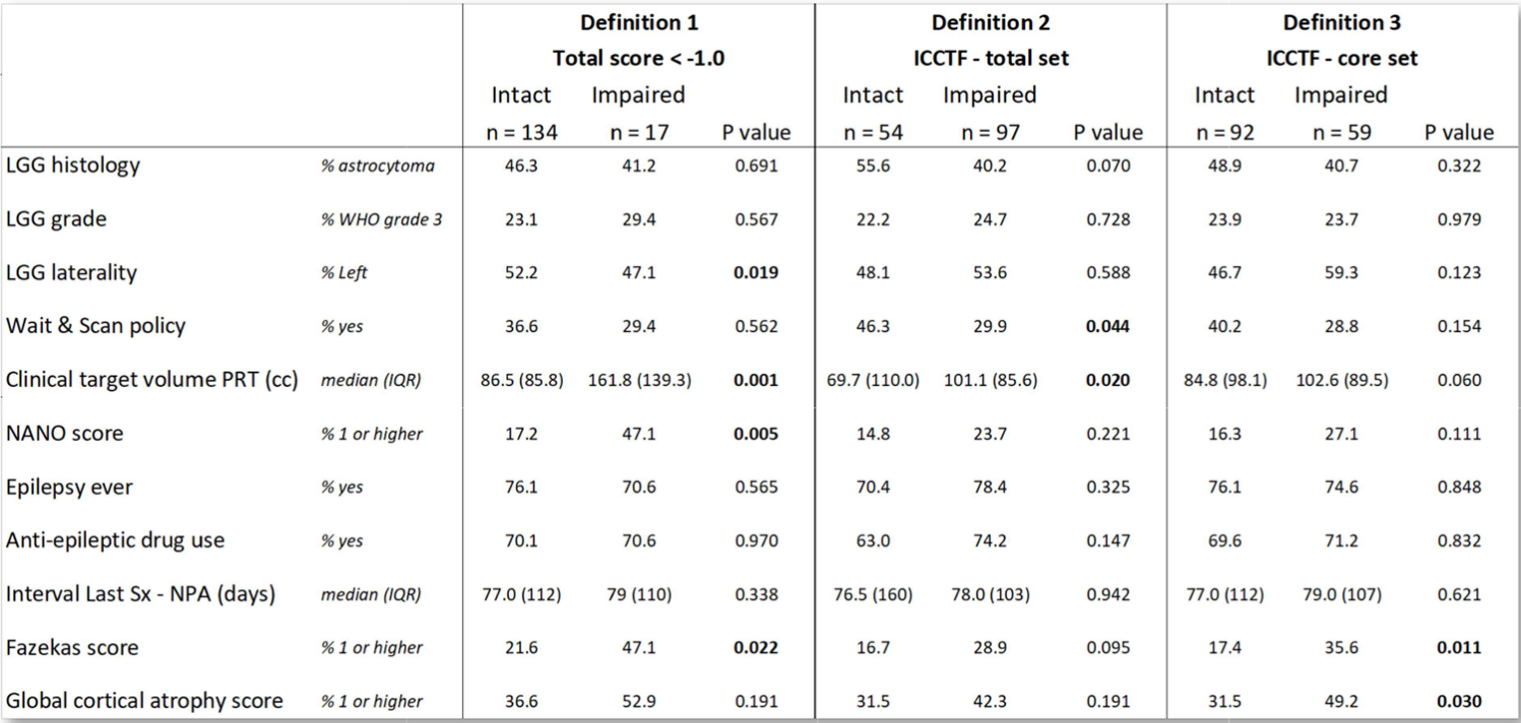
Differences in clinical factors between NCF impairment groups. Patients were indicated as impaired NCF based on 3 different definitions, and compared with intact patients. ICCTF = international cognition and cancer task force

### NCF domain scores and tumor lobe involvement

Table 4 shows that, after correction for multiple comparisons, patients with left temporal lobe involvement of the tumor displayed significantly lower scores on verbal memory, and patients with occipital lobe involvement of the tumor displayed significantly lower scores on processing speed.

**Table 4:**
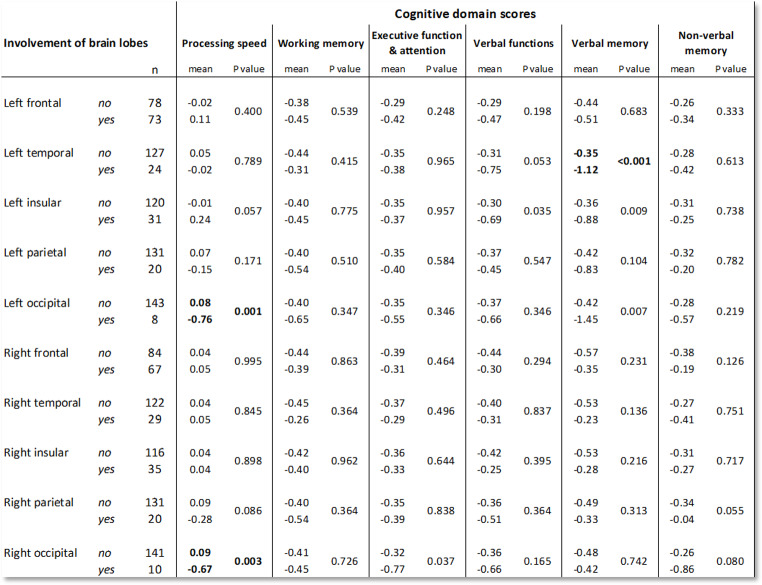
Differences between tumor location groups per cognitive domain. Bold results are significant after Bonferroni-Holm correction for multiple comparisons

## Discussion

This study is the first to provide a comprehensive description of baseline NCF, including variability of cognitive profiles and the incidence of impairments, in a large representative cohort of patients with LGG who are eligible for PRT because of their favorable prognosis. These patients are considered to have high chances for long term survival and to benefit most from the brain and NCF sparing capabilities of PRT compared to photon RT. The LGG patients in this study were selected for PRT following three nationally defined eligibility criteria indicating favorable prognosis [21]. However, neurocognitive status is not an ingredient of these criteria, and our data show that not all selected patients with favorable clinical characteristics have a favorable NCF profile.

We found that there was substantial variability in NCF profiles ranging from completely intact to overall impaired. The average composite total and domain NCF scores were significantly lower than the norm-scores of the healthy population, indicating the effects of tumor and previous treatment. However, when assessed individually, the majority of the patients (66.2%) had a favorable NCF profile without cognitive domain impairments. On the other end of the spectrum, there was a subgroup of patients (11.3%) performing on an overall impaired level. Cognitive impairments were most frequently observed in the memory domains. Relevant clinical and radiological covariates associated with impaired NCF identified in this study are a treatment history with Wait & Scan policy, left temporal or occipital lobe tumor involvement, tumor laterality, tumor volume, neurological function as assessed by NANO and the radiological appearance of the surrounding brain as assessed by Fazekas and GCA score. Wait & Scan policy is a strategy considered in patients with more favorable features, applied in 35.8% of patients in our cohort. At start of PRT, these patients did not perform worse than their counterparts, indicating that deferring the neuro-toxic effects of radio-chemotherapy did not come at the expense of a more unfavorable NCF in these patients. However, the changes in function over the Wait & Scan trajectory were not measured.

We found that working memory and verbal memory were most severely affected prior to PRT, in line with findings of Lemaitre et al. [12] of neurocognitive performance three months post-surgery. This may be a finding typically seen in patients with left-sided, mostly temporal LGG, in the early post-surgery phase that might improve over time [19]. Our finding of a relation with verbal memory between left temporal lobe tumor involvement and immediate post-surgery referral (without Wait & Scan policy) supports this postulation.

We aimed to identify the subgroup of patients performing overall on an unfavorable, impaired level. However, in literature no uniform definition for NCF impairment is used [23]. We compared 3 commonly applied definitions, and observed a very large difference in number of patients subsequently labeled as impaired. With definition 1 (total composite Z score < -1.0), a relatively small subset of patients (11.3%) was selected, that overall displayed the lowest performance and more diffuse NCF damage. Definition 2 (ICCTF criterium whole test battery) classified a much larger subset of patients (64.2%) as impaired. Definition 3 (ICCTF criterium on core test set), selected an intermediate number of patients (39.1%). Consequently, depending on the definition of NCF impairment applied, different associated clinical and radiological covariates were found. Definition 1 can be regarded as a rather crude method for LGG patients, especially when appraising the large heterogeneity of expressed NCF profiles. Definition 2 is not a very practical as extended test batteries are not likely being conducted in many other centers and may this method may provide an overestimation. In our opinion, definition 3 is the preferred method as it is a balanced alternative.

In this cohort of IDH mutated LGG patients, NCF impairment was not related to LGG subtype, nor to epilepsy associated factors, although these are considered important covariates for NCF [16]. Depending on the definition of NCF impairment used tumor size (CTV as surrogate), right-sided LGG location and neurological function (NANO score), were relevant factors. A new interesting finding in our study is the association between NCF impairment and the radiological aspect of the brain (Fazekas and GCA score). Despite the young age and limited comorbidities of patients, the prevalence of any sign of white matter damage or atrophy was relatively high: 24.5% and 38.4% respectively. These radiological indices could be a reflection of the patient’s vulnerability to neurotoxicity and/or the ability to respond and adapt to damage.

Furthermore, overall limited associations between tumor location and cognitive domain scores were found, for which the slow growth of LGG, the plasticity and adaptation of the brain over years in response, as well as the functional organization of the brain with underlying neuronal networks are likely explanations [8, 26, 27]. However, we still found associations between: (1) the left temporal lobe and verbal memory; and (2) the occipital lobe and processing speed. The first finding is in line with other studies [19, 28]. Visual field deficits could be a good explanation for the second finding. In our study processing speed is evaluated by primarily visual tasks, and patients with visual field deficits (NANO visual field sub-score 1 or higher, *n* = 8) displayed significantly lower processing speed (mean composite Z score − 0.56 versus 0.09, *p* = 0.05), and patients with occipital tumors were more likely to have visual field deficits.

The strength of this study is the large, homogeneous sample size and the very high compliance rate with the NPA of our prospective registration program (net compliance rate 94%), reflecting the relevance for patients and caregivers. Therefore, these findings can be regarded as real-world data. However, it is important to note that the eligibility criteria for PRT as well as the timing of RT treatment in the disease course of LGG patients are variable between countries.

A limitation of our study is the lack of NPA pre-surgery and during Wait & Scan trajectory. Therefore, we cannot report on the pre-diagnosis functioning of the patients or the duration of impairments. The qualitative scoring of tumor lobe involvement that was used in this study, is a practical method that can be easily adopted in clinical practice. However, as this is a very crude way to assess tumor involvement, a voxel-based methodology might be more sensitive to detect correlations between tumor location and specific NCF impairments [28]. In this study we did not measure pre-operative tumor volumes. In the patients with Wait & Scan policy and/or multiple resections it is not straight forward to measure a pre-surgery volume. Therefore, we used the PRT CTV as a surrogate in this study.

Our baseline data show that overall patients considered eligible for PRT have a favorable NCF profile, but also that a subgroup has NCF impairment. It is yet unknown what the effects of PRT will be for this group, differentiated across impairments levels or cognitive domains. To date, there is still very limited knowledge on the relation between RT dose and NCF changes over time [16, 17, 29]. Therefore, well-organized clinical registration studies to obtain high-quality longitudinal NCF data after PRT are inevitable to move forward [25]. This knowledge can drive future RT technique optimization, and might have an important value for improving clinical outcome [30, 31].

## Conclusion

This study provides an in-depth examination of NCF in a large homogeneous cohort of selected LGG patients at initiation of PRT. Overall, the patients display heterogenous NCF profiles with limited impairments. Consequently, there is a compelling case for minimizing the unnecessary RT dose to the non-involved brain in these patients. Relevant clinical and radiological covariates associated with NCF identified in this study are a treatment history with Wait & Scan policy, left temporal or occipital lobe tumor involvement, tumor laterality, tumor volume, neurological function as assessed by NANO and the radiological appearance of the surrounding brain as assessed by Fazekas and GCA score. Establishing a comprehensive baseline for NCF prior to PRT are an imperative first step in future efforts to evaluate and exploit the NCF preserving effects PRT.

## Electronic supplementary material

Below is the link to the electronic supplementary material.

Supplementary Material 1

## Data Availability

No datasets were generated or analysed during the current study.

## Abbreviations

IDH: Isocitrate Dehydrogenase
LGG: Lower Grade Glioma
RT: Radiotherapy
NCF: Neurocognitive Function
PRT: Proton Radiotherapy
UMCG: University Medical Center Groningen
NANO: Neurological Assessment in Neuro-Oncology
AED: Anti-Epileptic Drug
NPA: Neuropsychological Assessment
CTV: Clinical Target Volume
GCA: Global Cortical Atrophy
ICCTF: International Cognition and Cancer Task Force
EPTN: European Particle Therapy Network
RAVLT: Rey’s auditory Verbal Learning Test
COWAT: Controlled Oral Word Association Test
TMT: Trail Making Test
IQR: Inter-Quartile Range
