# Supplementary material for: Neurocognitive function in lower grade glioma patients selected for proton radiotherapy: real-world data from a prospective cohort study
Source: J Neurooncol. 2025 Feb 20;173(1):147–56. doi: 10.1007/s11060-025-04973-7 (PMC12041185; doi:10.1007/s11060-025-04973-7)
Supplement: Supplementary file 1 — Supplementary Material 1 [file 11060_2025_4973_MOESM1_ESM.docx]

**Supplementary Tables**

**Supplementary Table 1.** Prospective data registration program for patients with lower grade glioma referred for proton radiotherapy to the University Medical Center Groningen, the Netherlands. Prior to start of proton radiotherapy and every 2.5 years thereafter, patients are invited for a multi-disciplinary assessment on site.

**
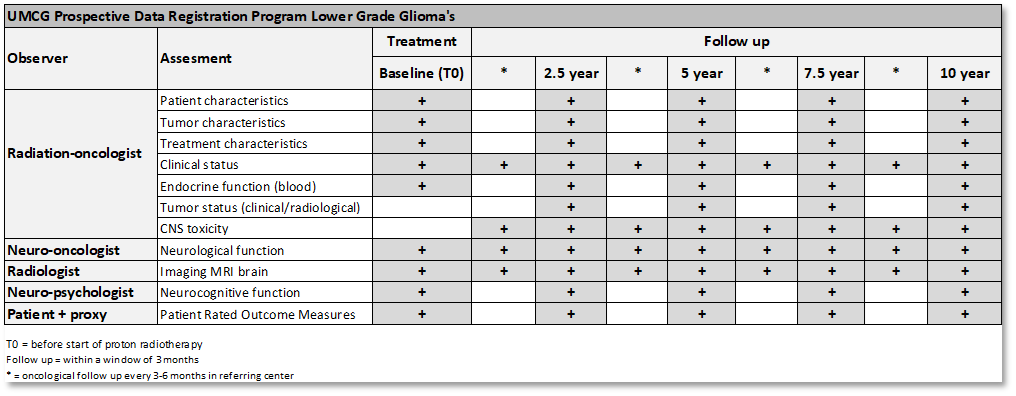
**

**Supplementary Table 2.** Associations of cognitive domain composite Z-scores with clinical variables. Bold results are significant after Bonferroni-Holm correction for multiple comparisons.

**
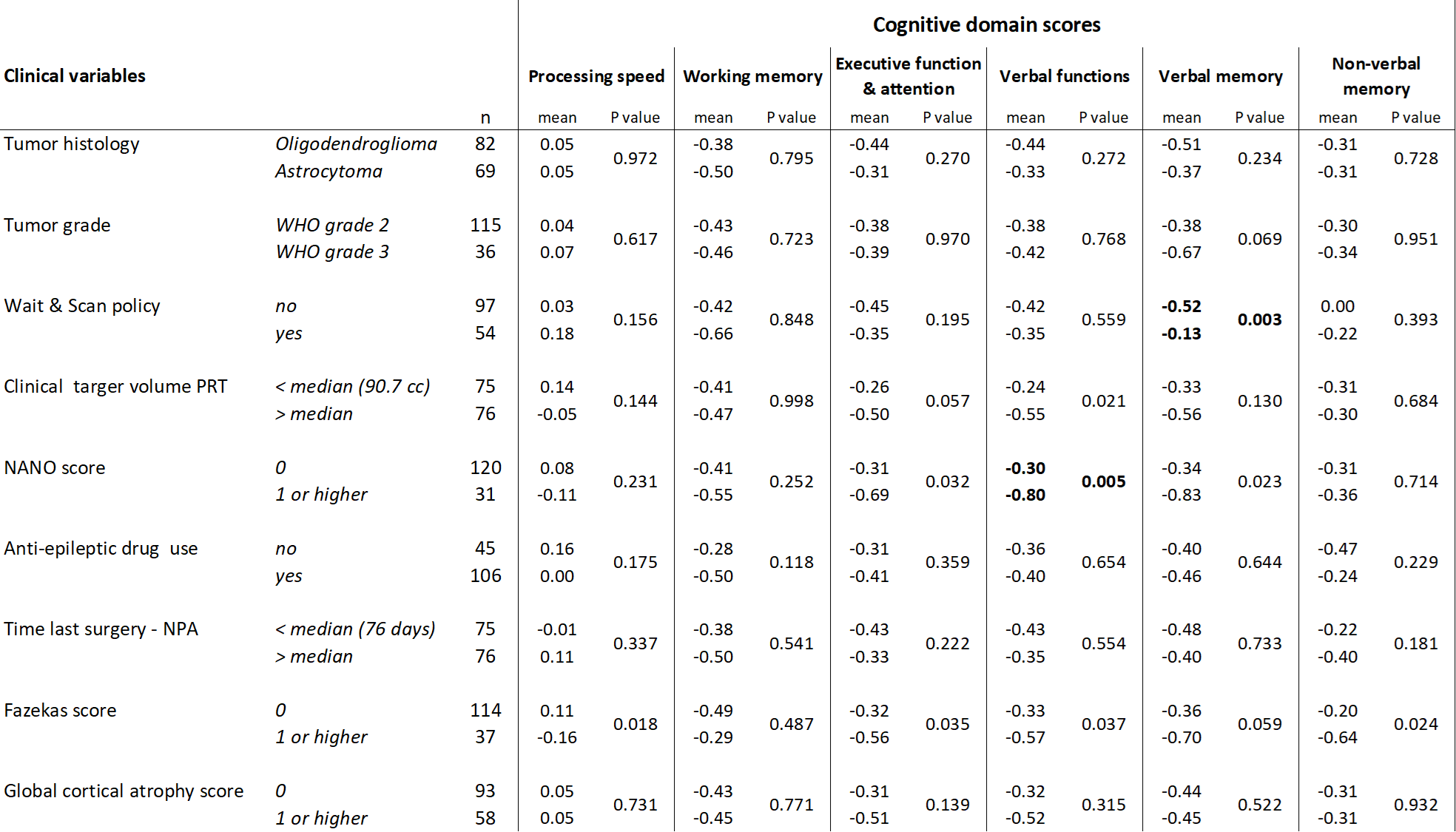
**
